# Supplementary material for: Multi-strategy evolutionary games: A Markov chain approach
Source: PLoS One. 2022 Feb 17;17(2):e0263979. doi: 10.1371/journal.pone.0263979 (PMC8853582; doi:10.1371/journal.pone.0263979)
Supplement: Attachment — Submitted filename: Response to Reviewers.pdf [file pone.0263979.s001.pdf]

Dear Editor,

Thank you for your communication of January 17, 2022. We are submitting the revised version of our paper (PONE-D-21-40483) to PLOS one. The paper has been considerably revised and improved in response to the comments and suggestions raised by the referee. We would like to thank the referees for their valuable comments and suggestions. We hope that our modifications fulfill their criticisms/comments.

Reviewer 1:

Here we respond to comments raised by the referee:

Markov chain approach, although the authors said a 'theoretical' approach, provides the dynamics of a time-discrete system. In this sense, the methodology can be said an 'approximation' of an inherently time continuous system. I would like to suggest the authors to note this remark somewhere to be appropriate in the revised MS.

In introduction we mentioned that the method can be considered as approximation of a time continuous system.

RPS game is a quite good template which may attract quite a few people dealing with EGT. In this sense, I suggest the authors to more deeply review some up-coming fruits on RPS game for recent years by citing some of them.

We reviewed some up-coming papers on RSP game and cited some of them.

Besides RPS game, a question coming to mind is why the authors not trying to 2 by 2 game including Prisoner's Dilemma (PD), Chicken, Stag Hunt (SH) and Trivial game classes that have been regarded as the most typical template in the field of EGT. More importantly, the issue of social dilemma represented by either PD, Chicken, or SH might be more pressing in terms of application than RPS game. Although I wouldn't go as far as to say that the authors should do with further series of Multi Agent Simulations (MASs), they should mention something somewhere (perhaps, at the end of conclusive remark) that there is wide possibility of application not only RPS game but also archetype 2 by 2 symmetric games by citing recent some review papers or books.

We added explanations to conclusion and we think that it is much better now.

Reviewer 2:

To evaluate implementation and soundness of our work we put our codes on Github.
